# Coal Workers’ Pneumoconiosis–Associated Deaths — United States, 2020–2023

**DOI:** 10.15585/mmwr.mm7441a1

**Published:** 2025-12-18

**Authors:** Jacek M. Mazurek, Katelynn E. Dodd, Girija Syamlal, David J. Blackley, David N. Weissman

**Affiliations:** 1 Respiratory Health Division, National Institute for Occupational Safety and Health, CDC.

**Summary**

**What is already known about this topic?**

Coal workers’ pneumoconiosis (CWP) is an occupational lung disease caused by inhaling coal mine dust. Although the disease is preventable, new cases and CWP-associated deaths continue to occur.

**What is added by this report?**

In this exploratory analysis of 2020–2023 national cause-of-death data, 1,754 deaths associated with CWP among U.S. residents aged ≥15 years were reported (age-adjusted CWP death rate = 1.3 per 1 million). Increased risk for CWP-associated death was observed among workers in the mining industry and among construction and extraction workers.

**What are the implications for public health practice?**

The continuing occurrence of CWP-associated deaths underscores the potential value of a comprehensive prevention program (control of exposures to coal mine dust, early disease detection, and medical care) and supports potential benefits of ongoing surveillance.

## Abstract

Coal workers’ pneumoconiosis (CWP) is a preventable, progressive occupational lung disease caused by inhaling respirable coal mine dust, a complex mixture commonly containing coal, crystalline silica, and other silicate minerals. Early pneumoconiosis can be asymptomatic, but advanced disease often leads to disability and premature death. To describe CWP-associated mortality among U.S. residents aged ≥15 years by industry and occupation, CDC conducted an exploratory analysis of National Vital Statistics System multiple cause-of-death data for 2020–2023, the most recent years that include information on decedents’ usual industry and occupation. During 2020–2023, CWP was listed on the death certificate of 1,754 decedents (age-adjusted CWP-associated death rate = 1.3 per 1 million). By industry group, the highest number of CWP-associated deaths occurred among workers in the mining industry (1,255). The highest proportionate mortality ratios (PMRs) were among persons employed in the mining industry (PMR = 50.0) and the construction and extraction occupations (6.2). Among workers employed in the mining industry, the highest PMR was among underground mining machine operators (164.6). The continuing occurrence of CWP-associated deaths underscores the potential value of a comprehensive prevention program (maintaining efforts to control occupational coal mine dust exposures, combined with early disease detection efforts and medical care) and supports potential benefits of ongoing surveillance.

## Introduction

Coal workers’ pneumoconiosis (CWP) is a preventable, progressive occupational lung disease caused by inhaling respirable coal mine dust, a complex mixture commonly containing coal, crystalline silica, and other silicate minerals (*1*,*2*). The disease is characterized by progressive scarring of lung tissue and declining lung function (*1*,*2*). Disease progression can occur after cessation of exposure (*2*,*3*). Early pneumoconiosis can be asymptomatic, but advanced disease often leads to disability and premature death. No specific treatment exists for CWP; patients with CWP receive supportive care. Lung transplantation can be offered to eligible patients with end-stage CWP; median posttransplant survival is 6.6 years (*2*,*4*). Controlling exposure to coal mine dust, combined with early disease detection efforts and medical care, are all important prevention methods to reduce morbidity and mortality associated with CWP (*1*,*2*,*5*,*6*). To describe CWP-associated mortality among U.S. residents aged ≥15 years by decedents’ usual industry and occupation (i.e., the industry and occupation the decedents engaged in for most of their working life) and to examine associations between CWP-associated deaths and industry and occupation, CDC conducted an exploratory analysis of 2020–2023 National Vital Statistics System (NVSS) multiple cause-of-death data.

## Methods

### Case Definition and Data Source

CWP-associated decedents were defined as persons whose death record listed the International Classification of Diseases, Tenth Revision, Clinical Modification code J60 (coal workers’ pneumoconiosis) as the underlying or a contributing cause of death.* The NVSS public-use multiple cause-of-death files include 13,098,787 records for U.S. resident decedents aged ≥15 years who died during 2020–2023, the most recent years with information on decedents’ usual industry and occupation. The NVSS files include decedents’ occupation and industry information coded through a collaboration with CDC’s National Institute for Occupational Safety and Health (NIOSH). The 2020–2022 occupation and industry information was coded to 2012 CDC Census Occupation and Industry codes, and the 2023 occupation and industry information was coded to 2018 CDC Census Occupation and Industry codes. Overall, 12,796,979 (97.7%) records included information on industry and occupation.

### Data Analyses

Death rates (number of deaths per 1 million persons aged ≥15 years) were based on annual postcensal population estimates as of July 1 of the corresponding year. Death rates were age adjusted by applying age-specific death rates to the 2000 U.S. Census Bureau standard population (Multiple Cause of Death 2018–2023 by Single Race). The state on the death record represents the decedent’s place of legal residence at the time of death.

For analyses, because of small numbers of observations in certain industry and occupation narratives, major industry categories were reduced from 22 to 21 groupings by combining military, miscellaneous, and unclassifiable categories; major occupation categories were reduced from 25 to 23 groupings by combining military, miscellaneous, unclassified, and homemakers categories. One occupation might be listed under multiple industries.

Proportionate mortality ratios (PMRs) (defined as the observed number of deaths with CWP in a specified industry or occupation, divided by the expected number of deaths with CWP) and 95% CIs (assuming Poisson distribution of the data) were calculated by industry and occupation categories. The expected number of deaths was the total number of deaths in an industry or occupation of interest multiplied by a proportion defined as the number of CWP-associated deaths in all industries or occupations, divided by the total number of deaths in all industries or occupations. PMRs were adjusted by 10-year age groups, sex, race, and ethnicity. PMR >1.0 or <1.0 indicates that more or fewer deaths than expected, respectively, were associated with the condition in a specified occupation or industry. PMRs were considered statistically significant if the 95% CI excluded 1.0.

An additional analysis of PMRs was performed by detailed occupation for the decedents whose longest-held job was in the mining industry. For this analysis, to address the updated occupation coding system, detailed 2012 occupation codes were converted to the 2018 coding scheme (Industry and Occupation Code Lists and Crosswalks | US Census Bureau).

Analyses were conducted using CDC Wide-ranging ONline Data for Epidemiologic Research (WONDER) and SAS software (version 9.4; SAS Institute). This activity was reviewed by CDC, deemed not research, and was conducted consistent with applicable federal law and CDC policy.^†^

## Results

### CWP-Associated Deaths and Death Rates

During 2020–2023, a total of 1,754 deaths with CWP listed as the underlying (576; 32.8%) or a contributing (1,178; 67.2%) cause of death were identified among U.S. residents aged ≥15 years (Table 1), accounting for 0.01% of 13,098,787 deaths from all causes. Overall, the annualized age-adjusted CWP-associated death rate was 1.3 per 1 million persons. During 2020–2023, the annual number of CWP-associated deaths increased from 370 (death rate = 1.1 per million) in 2020 to 462 (1.4) in 2023. Among CWP-associated deaths, 1,382 (78.8%; age-specific death rate = 6.1 per million persons) occurred among persons aged ≥65 years, 1,663 (94.8%; age-adjusted death rate = 2.8) among males, 1,692 (96.5%; 1.5) among White persons, and 1,715 (97.8%; 1.4) among non-Hispanic persons.

**TABLE 1:** Characteristics of decedents with coal workers’ pneumoconiosis and age-adjusted coal workers’ pneumoconiosis–associated death rates among persons aged ≥15 years — United States, 2020–2023

Characteristic	CWP-associated deaths,* no. (%)	Death rate^†^ (95% CI)
**Total**	**1,754 (100.0)**	**1.3 (1.3–1.4)**
**Age group, yrs^§^**		
15–44	62 (3.5)	0.1 (0.1–0.2)
45–64	310 (17.7)	0.9 (0.8–1.0)
≥65	1,382 (78.8)	6.1 (5.7–6.4)
**Sex**		
Female	91 (5.2)	0.1 (0.1–0.2)
Male	1,663 (94.8)	2.8 (2.7–3.0)
**Race** ^¶^		
Black or African American	45 (2.6)	0.3 (0.2–0. 5)
White	1,692 (96.5)	1.5 (1.5–1.6)
Multiple races	17 (1.0)	—**
**Ethnicity**		
Hispanic or Latino	39 (2.2)	0.3 (0.2–0.4)
Non-Hispanic	1,715 (97.8)	1.4 (1.4–1.5)
**Year**		
2020	370 (21.1)	1.1 (1.0–1.2)
2021	459 (26.2)	1.4 (1.3–1.5)
2022	463 (26.4)	1.4 (1.2–1.5)
2023	462 (26.3)	1.4 (1.2–1.5)
**State of residence**		
≥10 deaths^††^		
Kentucky	517 (29.5)	27.9 (25.5–30.4)
West Virginia	334 (19.0)	38.4 (34.2–42.5)
Virginia	281 (16.0)	8.1 (7.2–9.1)
Pennsylvania	164 (9.4)	3.3 (2.8–3.9)
Tennessee	82 (4.7)	2.9 (2.3–3.6)
Colorado	48 (2.7)	2.3 (1.7–3.1)
Alabama	21 (1.2)	1.0 (0.6–1.5)
South Carolina	20 (1.1)	0.9 (0.5–1.4)
Ohio	41 (2.3)	0.9 (0.6–1.2)
Illinois	30 (1.7)	0.6 (0.4–0.8)
Texas	43 (2.5)	0.4 (0.3–0.6)
Florida	25 (1.4)	0.3 (0.2–0.4)
Indiana	18 (1.0)	—**
Wisconsin	16 (0.9)	—**
New Mexico	15 (0.9)	—**
North Carolina	14 (0.8)	—**
Utah	14 (0.8)	—**
All other states^§§^	71 (4.0)	0.1 (0.1–0.2)

**Source**: CDC Wide-ranging ONline Data for Epidemiologic Research (WONDER) online databases, Multiple Cause of Death, 2018-2023, Single Race Request.

**Abbreviations**: AI/AN = American Indian or Alaska Native; A/PI = Asian or Pacific Islander; CWP = coal workers’ pneumoconiosis; NCHS = National Center for Health Statistics.

* Death records with *International Classification of Diseases, Tenth Revision, Clinical Modification* multiple cause-of-death code J60 (coal workers’ pneumoconiosis).

^†^ Age-adjusted CWP-associated death rates (deaths per 1 million persons) were calculated by applying age-specific death rates to the 2000 U.S. Census Bureau standard population age distribution. Multiple Cause of Death 1999-2020

^§^ Age-specific CWP-associated deaths per 1 million persons. The minimum age to work in a mine is 18 years; however, Bureau of Labor Statistics data indicate that during 2024, approximately 7,000 workers aged 16–19 years were employed in the mining, quarrying, and oil and gas extraction industries, mainly in support activities for mining. No CWP-associated deaths were reported among persons aged 15–19 years.

^¶^ Race and Hispanic origin are reported separately on the death certificate. The AI/AN race category includes North, Central, and South American Indians, Eskimos, and Aleuts. The A/PI race category includes Chinese, Filipino, Hawaiian, Japanese, and other A/PIs (Multiple Cause of Death 2018-2023 by Single Race). Race and ethnicity on death certificates might be misclassified. Interpretation of current mortality data relies on the most recent linkage-based evaluation conducted by CDC’s NCHS for deaths occurring through 2011. The evaluation study found that race and Hispanic origin reporting is highly accurate for non-Hispanic White and non-Hispanic Black or African American decedents, and generally accurate for Hispanic or Latino (Hispanic) and A/PI decedents. However, the study identified substantial underreporting among AI/AN decedents, with approximately 40% of AI/AN deaths misclassified overall and 33% misclassified among non-Hispanic AI/AN decedents. As a result, the quality of race and Hispanic-origin reporting for the AI/AN population might be poor, and mortality for this group might be underestimated. NCHS, Series 2, no 172

** Dashes indicate death rate is unreliable. NCHS considers death rates based on counts of fewer than 20 deaths unreliable. A death rate based on fewer than 20 deaths has a relative SE ≥23% and is considered statistically unreliable. Multiple Cause of Death 2018-2023 by Single Race

^††^ NCHS data use restrictions preclude death counts of fewer than 10, as well as death rates based on counts of fewer than 10.

^§§^ States with fewer than 10 CWP-associated deaths: Alaska, Arizona, Arkansas, California, Connecticut, Georgia, Iowa, Kansas, Louisiana, Maryland, Massachusetts, Michigan, Minnesota, Mississippi, Missouri, Montana, Nebraska, Nevada, New Jersey, New York, Oklahoma, Oregon, South Dakota, Washington, and Wyoming. No CWP-associated deaths occurred in Delaware, the District of Columbia, Hawaii, Idaho, Maine, New Hampshire, North Dakota, Rhode Island, or Vermont.

During 2020–2023, 17 states reported at least 10 CWP-associated deaths^§^ among persons aged ≥15 years. Deaths in four states (Kentucky [517; annualized age-adjusted CWP-associated death rate = 27.9 per million persons], West Virginia [334; 38.4], Virginia [281; 8.1], and Pennsylvania [164; 3.3]) accounted for 1,296 (73.9%) of all CWP-associated decedents.

### Industry

Industry and occupation data were available for 1,748 (99.7%) of 1,754 CWP-associated deaths. Among the 21 industry groups, the highest number of CWP-associated deaths occurred among persons employed in the mining industry for most of their working life (1,255; 71.8%), followed by the construction (111; 6.4%) and manufacturing (68; 3.9%) industries (Table 2). PMR among persons working in the mining industry was 50.0, indicating that the observed number of CWP-associated deaths was significantly higher than the expected number of CWP-associated deaths.

**TABLE 2:** Industries and occupations with coal workers’ pneumoconiosis–associated deaths and proportionate mortality ratio among persons aged ≥15 years — selected U.S. jurisdictions, 2020–2023

Characteristic	No. of deaths from all causes	CWP-associated deaths,* no. (%)	PMR^†^ (95% CI)
**Total^§^**	**12,796,979**	**1,748 (100)**	**—**
**Industry^¶^**			
Agriculture	270,274	15 (0.9)	0.3 (0.2–0.5)
Mining	91,110	1,255 (71.8)	50.0 (47.3–52.9)**
Construction	946,095	111 (6.4)	0.5 (0.4–0.6)
Manufacturing	1,568,911	68 (3.9)	0.3 (0.2–0.3)
Retail trade	891,149	18 (1.0)	0.2 (0.1–0.3)
Transportation and warehousing	668,960	42 (2.4)	0.3 (0.2–0.4)
Administrative and support and waste management and remediation services	302,018	21 (1.2)	0.5 (0.3–0.8)
Health care and social assistance	1,118,129	21 (1.2)	0.3 (0.2–0.5)
Accommodation and food services	467,120	18 (1.0)	0.4 (0.3–0.7)
Other services (except public administration)	598,929	41 (2.3)	0.5 (0.3–0.6)
Public administration	598,745	21 (1.2)	0.2 (0.1–0.3)
Military, miscellaneous, or unclassifiable	2,744,071	79 (4.5)	0.4 (0.3–0.5)
All other industries^††^	2,531,468	38 (2.2)	—
**Occupation^¶^**			
Management	1,073,693	41 (2.3)	0.2 (0.1–0.3)
Business and financial operations	329,310	10 (0.6)	0.2 (0.1–0.4)
Community and social services	172,892	12 (0.7)	0.5 (0.3–1.0)
Protective service	227,043	13 (0.7)	0.3 (0.2–0.5)
Food preparation and serving related	372,610	12 (0.7)	0.5 (0.3–0.9)
Building and grounds cleaning and maintenance	385,595	37 (2.1)	0.8 (0.6–1.1)
Sales and related	884,097	19 (1.1)	0.1 (0.1–0.2)
Construction and extraction	854,943	1,273 (72.8)	6.2 (5.8–6.5)**
Installation, maintenance, and repair	448,690	43 (2.5)	0.4 (0.3–0.5)
Production	955,637	55 (3.1)	0.3 (0.3–0.5)
Transportation and material moving	897,877	96 (5.5)	0.6 (0.5–0.7)
Military, miscellaneous, unclassifiable, and homemakers	2,674,437	83 (4.7)	0.4 (0.4–0.6)
All other occupations^§§^	3,520,155	54 (3.1)	—

**Source**: National Vital Statistics System public use multiple cause files 2020–2023. Data Access - Vital Statistics Online | CDC

**Abbreviations**: CWP = coal workers’ pneumoconiosis; PMR = proportionate mortality ratio.

* Death records with *International Classification of Diseases, Tenth Revision, Clinical Modification* multiple cause-of-death code J60 (coal workers’ pneumoconiosis).

^†^ PMR was defined as the observed number of deaths with CWP in a specified occupation, divided by the expected number of deaths with CWP. The expected number of deaths was the total number of deaths in the occupation of interest multiplied by a proportion defined as the number of CWP-associated deaths in all occupations, divided by the total number of deaths in all occupations. CWP-associated PMRs were adjusted by 10-year age groups, sex, race, and ethnicity.

^§^ In 2020, 47 jurisdictions participated in the industry and occupation information coding program (Arizona, North Carolina, Rhode Island, and the District of Columbia did not participate). Iowa participated in the program in 2020, but the data were inconsistent with those from other jurisdictions and were excluded. In 2021, a total of 49 jurisdictions participated (Rhode Island and the District of Columbia did not participate). In 2022 and 2023, a total of 52 jurisdictions (50 states, New York City, and the District of Columbia) participated in the program. Industry and Occupation Data Mortality 2020

^¶^ Recorded on decedents’ death certificates as the industry associated with the occupation the person held for most of their working life (i.e., usual industry and occupation).

** A lower bound of the 95% CI level with a value >1.0 indicates a statistically significantly elevated PMR.

^††^ Industries with fewer than 10 CWP-associated deaths: utilities; wholesale trade; information; finance and insurance; real estate and rental and leasing; professional, scientific, and technical services; management of companies and enterprises; education services; and arts, entertainment, and recreation.

^§§^ Occupations with fewer than 10 CWP-associated deaths: computer and mathematical; architecture and engineering; life, physical, and social science; legal; education, training, and library; arts, design, entertainment, sports, and media; health care practitioners and technical; health care support; personal care and service; office and administrative support; and farming, fishing, and forestry.

### Occupation

Among 1,748 workers employed in the 23 occupation groups for most of their working life, the highest number of CWP-associated deaths occurred among persons working in construction and extraction occupations (1,273; 72.8% of CWP-associated deaths), followed by those in transportation and material moving occupations (96; 5.5%). PMR of 6.2 among construction and extraction workers indicated that the observed number of CWP-associated deaths was statistically significantly higher than the expected number of CWP-associated deaths. Among workers in the mining industry, the highest number of CWP-associated deaths occurred among underground mining machine operators (851; PMR = 164.6) followed by other extraction workers (252; PMR = 75.0) (Table 3).

**TABLE 3:** Occupations with coal workers’ pneumoconiosis–associated deaths and proportionate mortality ratio among persons aged ≥15 years employed in the mining industry — selected U.S. jurisdictions,* 2020–2023

Occupation in mining industry^†^	No. of deaths from all causes (N = 91,110)	CWP-associated deaths,^§^ no. (%) (n = 1,255)	PMR^¶^ (95% CI)
Underground mining machine operators**	16,218	851 (67.8)	164.6 (153.7–176.0)^††^
Other extraction workers^§§^	12,609	252 (20.1)	75.0 (66.1–84.9)^††^
Electricians	1,365	25 (2.0)	58.0 (37.6–85.7)^††^
Construction equipment operators	2,849	25 (2.0)	29.5 (19.1–43.5)^††^
Driver/sales workers and truck drivers	3,309	22 (1.8)	24.2 (15.2–36.7)^††^
First-line supervisors of construction trades and extraction workers	4,879	11 (0.9)	7.9 (3.9–14.1)^††^
All other occupations^¶¶^	49,881	69 (5.5)	5.3 (4.1–6.7)^††^

**Source**: National Vital Statistics System public use multiple cause files 2020–2023. Data Access - Vital Statistics Online | CDC

**Abbreviations**: CWP = coal workers’ pneumoconiosis; PMR = proportionate mortality ratio.

* In 2020, 47 jurisdictions participated in the industry and occupation information coding program (Arizona, North Carolina, Rhode Island, and the District of Columbia did not participate). Iowa participated in the program in 2020, but the data were inconsistent with those from other jurisdictions and were excluded. In 2021, a total of 49 jurisdictions participated (Rhode Island and the District of Columbia did not participate). In 2022 and 2023, a total of 52 jurisdictions (50 states, New York City, and the District of Columbia) participated in the program. Industry and Occupation Data Mortality 2020

^†^ Mining industry recorded on decedents’ death certificates as the industry associated with the occupation the person held for most of their working life. Industry and Occupation Code Lists & Crosswalks

^§^ Death records with *International Classification of Diseases, Tenth Revision, Clinical Modification* multiple cause-of-death code J60 (coal workers’ pneumoconiosis).

^¶^ PMR was defined as the observed number of deaths with CWP in a specified occupation, divided by the expected number of deaths with CWP. The expected number of deaths was the total number of deaths in the occupation of interest multiplied by a proportion defined as the number of CWP-associated deaths in all occupations, divided by the total number of deaths in all occupations. CWP-associated PMRs were adjusted by 10-year age groups, sex, race, and ethnicity.

** Continuous mining machine operators; roof bolters and mining; and underground loading and moving machine operators.

^††^ Statistically significantly higher PMR based on a lower bound of the 95% CI with a value >1.0.

^§§^ Rock splitters, quarry; helpers and extraction workers; and all other extraction workers, not elsewhere specified.

^¶¶^ Occupations with fewer than 10 CWP-associated deaths: general and operations managers; property, real estate, and community association managers; compliance officers; industrial engineers, including health and safety; mining and geological engineers, including mining safety engineers; occupational health and safety specialists and technicians; first-line supervisors of firefighting and prevention workers; security guards and gambling surveillance officers; animal caretakers; first-line supervisors of retail sales workers; construction laborers; derrick, rotary drill, and service unit operators, oil and gas; first-line supervisors of mechanics, installers, and repairers; heavy vehicle and mobile equipment service technicians and mechanics; industrial and refractory machinery mechanics; maintenance and repair workers, general; first-line supervisors of production and operating workers; machinists; welding, soldering, and brazing workers; stationary engineers and boiler operators; miscellaneous plant and system operators; inspectors, testers, sorters, samplers, and weighers; laborers and freight, stock, and material movers, hand; earth drillers, except oil and gas; and other material moving workers.

## Discussion

CDC previously examined pneumoconiosis mortality by industry and occupation for selected states for 1999–2018 and identified similar industries (i.e., the coal mining and construction industries) and occupations (i.e., mining machine operators) associated with CWP deaths (*7*). This report expands information on industries and occupations associated with CWP deaths and reinforces the value of analyzing mortality data by industry and occupation.

Although CWP deaths would be expected to be limited to the workers in the mining industry, they were also identified across a range of other industries. The identification of CWP-associated deaths among workers employed in industries other than mining might be explained, in part, by the recording of only one occupation that the decedent held for most of their working life (i.e., usual occupation) on the death certificate and its associated industry. Coal mine workers might have skills that are shared with other industries (such as construction) and might move between the coal mine and other industries because of mine operation closures, poor health, or other reasons. Workers might also leave other long-held jobs and enter the coal mine industry.

A previous CDC analysis found a declining trend in CWP-associated deaths from 1999 (1,002 deaths; age-adjusted rate = 4.7 per million) to 2018 (305; 1.0) (*7*). In this report, the annual number of CWP-associated deaths increased from 370 (death rate = 1.1 per million) in 2020 to 462 (1.4) in 2023. This increase in CWP-associated deaths is consistent with recent findings indicating an increase in CWP prevalence and its most severe form (progressive massive fibrosis, characterized by the development of large, dense, fibrotic masses in the lungs) among coal miners working underground, particularly in central Appalachia (*8*,*9*). These trends in CWP prevalence have been associated with exposure to coal mine dust with a high content of crystalline silica and other silicate minerals, specifically in operations involving mining thin coal seams or cutting rock to access coal (*6*,*10*).

In 2014, a revised standard^¶^ decreased the existing permissible limit of exposure to respirable coal mine dust, increased dust sampling frequency and monitoring by mine operators, extended medical surveillance to include surface coal miners, and expanded medical surveillance testing to include spirometry. A long latency is typical between first exposure to coal mine dust and diagnosis of CWP, with disease most frequently identified in miners with ≥25 years’ tenure (*1*,*2*). Because persons with CWP can live for many years after diagnosis, the latency until death is even longer. Thus, insufficient time would likely have elapsed for the changes in regulations to substantially affect the findings in this report. Continued monitoring of mortality trends, with attention to industry and occupation, is important to assess the effects of these changes.

### Limitations

The findings in this report are subject to at least six limitations. First, CWP reported on death certificates were not validated using medical records. Some CWP deaths might have been attributed to other chronic respiratory diseases caused by exposure to coal mine dust (e.g., silicosis or chronic obstructive pulmonary disease) (*1*,*2*). Thus, CWP deaths might have been overascertained or underascertained. Second, some decedents might have never received a diagnosis of CWP (*6*). Therefore, occupations well-known to be associated with CWP deaths (e.g., underground mining machine operator) (*8*) might be underreported. Third, complete work histories were not available to enable assessment of changes in employment. Death certificate data relating to the decedent’s usual occupation and industry might not always reflect jobs in which causative exposures occurred. Some miners might have changed their jobs within the mining industry (e.g., miners with radiographic evidence of CWP moving to a low-dust mine environment under part 90 rights**), changed to another industry, or left the workforce; thus, PMRs might not accurately represent occupational risk. Fourth, the state issuing a death certificate might not be the state in which the decedent’s exposures occurred. Fifth, mortality rates might not correctly represent CWP frequency. The rates were calculated using data on the general population that include those who are not at an occupational risk for developing CWP. Finally, small numbers of deaths among certain groups did not permit a more detailed characterization of CWP-associated deaths.

### Implications for Public Health Practice

The continuing occurrence of CWP-associated deaths underscores the potential value of a comprehensive prevention program, including control of exposures to coal mine dust, early disease detection, and medical care (*1*,*2*,*5*,*6*) and supports potential benefits of ongoing surveillance. CDC’s NIOSH Coal Workers’ Health Surveillance Program provides information on diseases caused by coal mine dust, offers health screening to miners, informs miners with developing pneumoconiosis about their rights to work in a low-dust environment of the mine, and monitors disease occurrence. Miners’ awareness and participation in the Coal Workers’ Health Surveillance Program and mine operators’ support for respiratory health screenings are essential parts of the prevention efforts to reduce CWP morbidity and mortality.

## Footnotes

* Underlying cause of death is defined as “the disease or injury which initiated the chain of morbid events leading directly to death, or the circumstances of the accident or violence which produced the fatal injury.” A contributing cause of death (other significant condition) is any disease, condition, or injury that contributed to death but was not part of the chain of morbid events leading to death. Each death certificate contains up to 20 multiple causes of death. Instructions for Classification of Underlying and Multiple Causes of Death - Section I - 2021

† 45 C.F.R. part 46; 21 C.F.R. part 56; 42 U.S.C. Sect. 241(d), 5 U.S.C. Sect. 552a, 44 U.S.C. Sect. 3501 et seq.

§ National Center for Health Statistics data use restrictions preclude presenting death counts of fewer than 10, as well as death rates based on counts of fewer than 10.

¶ The concentration limits for respirable coal mine dust in air were decreased from 2.0 mg/m^3^ to 1.5/m^3^ at underground and surface coal mines and from 1.0 mg/m^3^ to 0.5 mg/m^3^ for intake air at underground mines, as well as for miners who already have evidence of developing pneumoconiosis. In addition, mine operators were required to use a continuous personal dust monitor to continuously measure the concentration of respirable coal mine dust and provide sampling results at specific time intervals (optional for surface coal mines and certain other mines). Medical surveillance required to be offered at no cost to underground and surface miners includes periodic occupational history, symptom assessment, chest radiograph, and spirometry with results reported to miners. In addition, part 90 miner transfer rights were extended to surface miners who have evidence of pneumoconiosis. Lowering Miners’ Exposure to Respirable Coal Mine Dust, Including Continuous Personal Dust Monitors | Federal Register 2014

** Miners with pneumoconiosis are protected under part 90 of Title 30 of the Code of Federal Regulations.
